# The complete chloroplast genome of *Carex agglomerata* C. B. Clarke (Cyperaceae), an endemic species from China

**DOI:** 10.1080/23802359.2021.1984326

**Published:** 2021-10-05

**Authors:** Lu-Lu Xun, Fang-Bin Ding, Chen Chen, Pei-Liang Liu, Yuan Lu, Ya-Fu Zhou, Ya-Wei Zhang, Si-Feng Li

**Affiliations:** a Xi’an Botanical Garden of Shaanxi Province, Institute of Botany of Shaanxi Province, Shaanxi Engineering Research Centre for Conservation and Utilization of Botanical Resources, Xi’an, Shaanxi, China; b College of Life Sciences, Northwest University, Xi’an, Shaanxi, China; c Baoji Animal Husbandry and Veterinary Center, Baoji, Shaanxi, China

**Keywords:** *Carex agglomerata*, chloroplast genome phylogenetic analysis sequencing

## Abstract

*Carex agglomerata* C. B. Clarke is a sedge with excellent ornamental characters, it is an important ecosystem stabilizer. Here we report the complete chloroplast genome of *C. agglomerata* to provide a foundation for further phylogenetic studies on the Cyperaceae. The chloroplast (cp) genome is 184,157 bp in size and consists of a large single-copy (LSC) region 106,654 bp in length, a small single-copy (SSC) region of 36,099 bp, two inverted repeats (IR) regions each 20,702 bp. The total GC content of the cp genome is 33.9% with the LSC, SSC, and IR regions 32, 32.5, and 42.9%, respectively. The cp genome contains 128 genes, including 80 protein-coding, 40 tRNA, and eight rRNA genes. The phylogenetic analysis showed *C. agglomerata* is in a clade with *Carex neurocarpa* Maxim and *Carex siderosticta* Hance. This study provides a basis for further phylogenetic studies of Carex.

The genus *Carex* is an important ecological taxon and is widely distributed in diverse environments ranging from the tundra, dry sand prairies, wetlands, and forests (Gadallah and Jefferies 1995). Species classified in *Carex* also play important ecological roles and provide food for animals (Gadallah and Jefferies 1995; Shrestha et al. 2012). In addition, some species have medicinal or nutritional properties (Roy et al. 2012). Many sedges have been widely used in gardens and new varieties have been bred due to their excellent ornamental properties (Darke 2007). Species classified to this genus are difficult to distinguish because of the morphological variability. Molecular-level evidence is thus required in order to distinguish species and provide an accurate systematic classification and evolutionary history. One of these species, *Carex agglomerate*, exhibits excellent ornamental features and is distributed in forests on mountain slopes and wet habitats in valleys at altitudes 1200–3000 m in Gansu, Qinghai, Shaanxi, and Sichuan (Dai et al. 2010). This species is also cultivated as ornamental grass and is used in gardens. Genetic studies of *C. agglomerata* are limited. SSR markers (Liu et al. 2021) showed a sister relationship between *C. agglomerata* and *Carex dimorpholepis* Steud. However, the complete chloroplast genome *C. agglomerata* has yet to be deciphered. Chloroplast genomes are highly conserved in sequence and structure and are of great importance in species identification and evolutionary studies (Zeng et al. 2017). This study determined the complete genome of *C. agglomerata* to contribute to the systematics and evolutionary history of the Cyperaceae.

Leaf tissue of *C. agglomerata* was collected from Ningshan County, Shaanxi province, China (108°29′40″E, 33°28′29.71″N, 2162 m). The voucher specimen was deposited at XBGH [the Herbarium of Xi’an Botanical Garden (HDT022, http://www.xazwy.com/, Bin Li, lbwj@163.com)]. Total genomic DNA was extracted using the CTAB method (Doyle 1987) and sequenced on Illumina Hi-Seq 2500 platform. In total, 3.16 Gb of raw data were obtained (NCBI Sequence Read Archive accession number SRR13414282). The raw reads were filtered using default settings in Trimmomatic v.0.33 (Bolger et al. 2014). The program NOVOPlasty (Dierckxsens et al. 2017) was used to de novo assemble the chloroplast genome. The program PGA (Qu et al. 2019) and Geneious v 11.0.2 (Kearse et al. 2012) performed the annotation followed by manual adjustments. The cp genome sequence was submitted to NCBI GenBank under accession number MT795185.

The cp genome of *C. agglomerata* is 184,157 bp in length and has a typical quadripartite structure, containing two IR regions 20,702 bp each, separated by an LSC region that is 106,654 bp and an SSC region of 36,099 bp. The total GC content is 33.9%, and the corresponding values of the LSC, SSC, and IR region are 32, 32.5, and 42.9%, respectively. This chloroplast genome has 128 functional genes, including 80 protein-coding (PCGs), 40 tRNA, and eight rRNA. The IR regions have four protein-coding genes (*ndhB*, *rpl2*, *rps12*, and *rps7*), five tRNA genes, and four rRNA genes. Sixteen genes (*atpF*, *ndhA*, *ndhB*, *petB*, *petD*, *rpl2*, *rpl16*, *rpoC1*, *rps12*, *rps16*, *trnA*, *trnG*, *trnI*, *trnK*, *trnL*, and *trnV*) contain one intron, and *ycf3* has two introns.

Ten other cp genomes of Cyperaceae and Poaceae from GenBank were used to determine the phylogenetic location of *C. agglomerata* in Cyperaceae, designating *Setaria viridis* as the out-group (Figure 1). The complete chloroplast sequences were aligned using MEGA 7.0 (Kumar et al. 2016) and the phylogenetic analysis was performed using the maximum likelihood (ML) method with 1000 bootstrap replicates was built by this program. The data showed that *C. agglomerata* was in a clade with *Carex neurocarpa* and *Carex siderosticta*. These findings are consistent with the results of SSR markers (Liu et al. 2021). The data are useful for future investigations of chloroplast genome evolution and phylogenetic research in the genus *Carex.*

**Figure 1.**
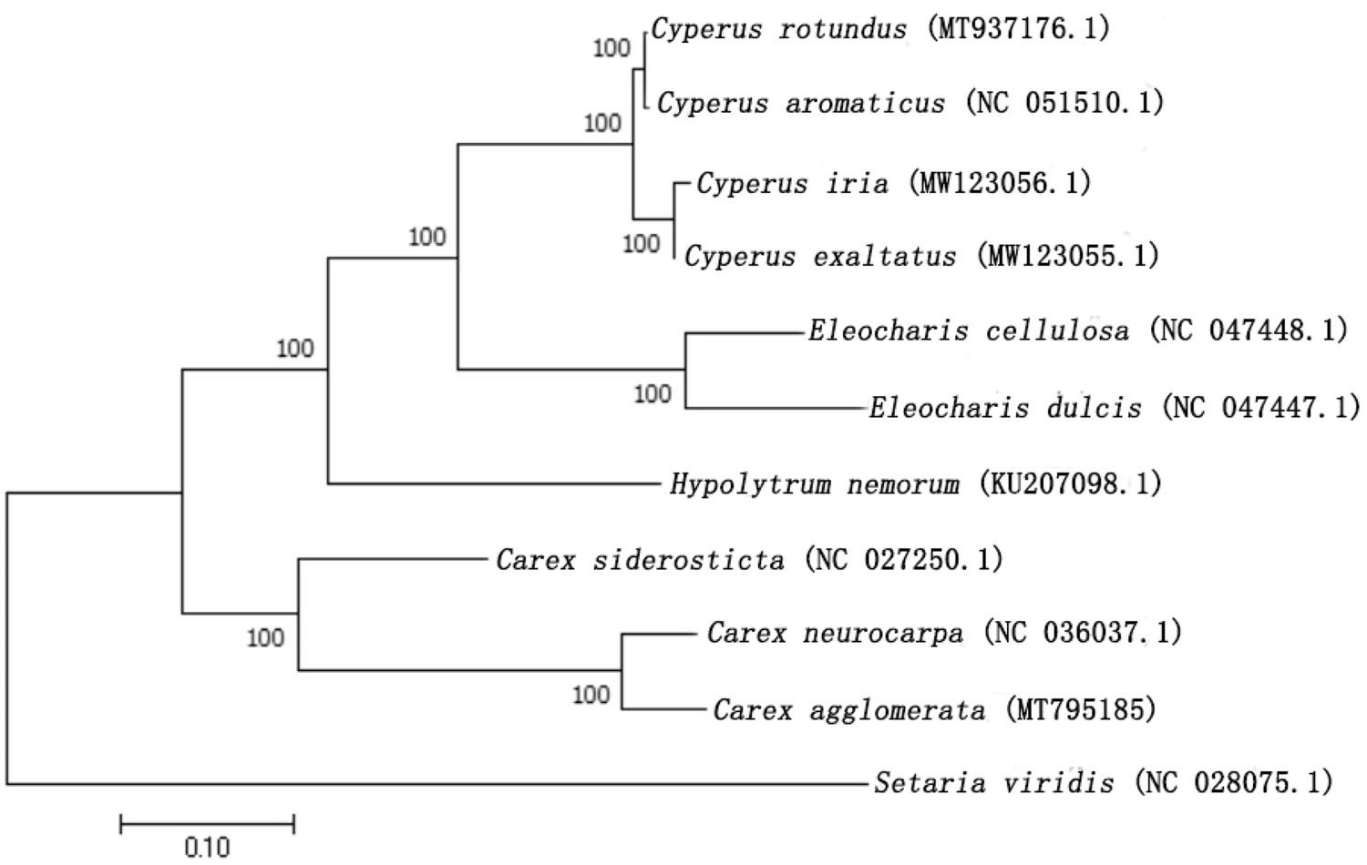
The phylogenetic tree was constructed with chloroplast genome sequences of 11 species within the Cyperaceae and Poaceae family and *Setaria viridis* as an outgroup by MEGA 7.0.

## Data Availability

The data that support the findings of this study are available in NCBI GenBank (https://www.ncbi.nlm.nih.gov) with accession number MT795185. The associated BioProject, SRA, and Bio-Sample numbers are PRJNA691435, SRR13414282, and SAMN17255546, respectively.
